# Screen Media Use Affects Subcortical Structures, Resting-State Functional Connectivity, and Mental Health Problems in Early Adolescence

**DOI:** 10.3390/brainsci13101452

**Published:** 2023-10-12

**Authors:** Xu He, Jiaxin Hu, Mengyun Yin, Wei Zhang, Boyu Qiu

**Affiliations:** 1 School of Health Management, Guangzhou Medical University, Guangzhou 510180, China; hexu@m.scnu.edu.cn; 2 Key Laboratory of Brain, Cognition and Education Sciences, South China Normal University, Guangzhou 510631, China; 3 School of Psychology, South China Normal University, Guangzhou 510631, China; 4 School of International Culture, South China Normal University, Guangzhou 510631, China

**Keywords:** screen media use, mental health problems, longitudinal data, subcortical structure, resting-state functional connectivity

## Abstract

The association between excessive screen media use and mental health problems has attracted widespread attention. The literature to date has neglected the biological mechanisms underlying such a relationship and failed to distinguish between different types of screen media activities. A sample from the Adolescent Brain and Cognitive Development study was used in the present study to elucidate the longitudinal associations between specific types of screen media use, brain development, and diverse mental health problems. The results showed that different types of screen media use have differentiated associations with mental health problems, subcortical volume, and cortical–subcortical connectivity. Specifically, more passive media use was associated with increased rule-breaking behavior, while more video game playing was associated with increased withdrawn/depressed symptoms. In addition, more social media use was associated with a reduced volume of the hippocampus, caudate, and thalamus proper. More research is needed to examine the differential effects of screen media use on neurodevelopmental processes and mental health problems across adolescence.

## 1. Introduction

Time spent on screen media such as social media, gaming, and TV has substantially increased for children and adolescents over the last two decades [1,2]. Nagata et al. [3] reported that the average daily screen time was 7.7 h per day in a national sample of adolescents (*N* = 5412) during the COVID-19 pandemic, which is about twice as much as the pre-pandemic estimates (3.8 h per day). A growing body of studies suggested that extensive exposure to screen media in youth might have a profound and lasting impact on mental health problems [4,5,6]. For example, Babic et al. [7] reported that changes in total recreational screen time were negatively associated with psychological well-being among adolescents. In a nationally representative sample of U.S. adolescents (*N* = 506,820), Twenge et al. [8] found that adolescents who reported more screen media use were significantly more likely to have high depressive symptoms. Anxiety symptoms have also been found to be associated with increases in screen media use [9].

However, this hypothesis was primarily based on the findings of cross-sectional studies. A systematic review of thirty-five longitudinal studies suggested that the impact of screen media use on mental health problems was negligible or small [10]. Based on the results of a meta-analysis including 33 recent studies published between 2015 and 2019, Ferguson et al. [11] indicated that there is no robust evidence to support the association between screen media use and mental health problems. The mixed results might be due to these studies failing to distinguish between different types of screen media activities, which vary in many factors, including interactivity (television watching is mostly a passive activity, while video game playing is interactive), time spent (social media is more likely to interfere with individuals’ daily lives), and social interaction (social media more often involves communication with people). A cross-sectional study has indicated that the association between screen media use and mental health differed by type of activity [12], with time spent on social media being more strongly associated with depressive symptoms than watching television. As cross-sectional design cannot afford conclusions about the directionality of effects, as well as temporal relationships, it is necessary to replicate the findings of the association between different types of screen media use and mental health problems using a longitudinal design.

At present, the biological mechanisms underlying the relationship between screen media use and mental health problems remain largely unclear. Cognitive neuroscience studies have demonstrated that brain areas of the affective system undergo extensive structural changes and functional reorganization during adolescence [13]. The ongoing brain development of adolescents makes them specifically reactive to emotion-arousing screen media [14], such as playing violent video games and experiencing online social rejection. For example, Quaglieri et al. [15] reported that emotional dysregulation was significantly and positively correlated with internet addiction and problematic social media use. There is also empirical evidence that emotional dysregulation increases the risk of a wide range of mental health problems in adolescence [16,17]. Therefore, additional work is warranted to test whether the aberrant development of emotion-related brain regions could serve as a mediator of the association between screen media use and mental health.

Previous studies indicated that the structural and functional development of subcortical brain regions involved in emotion generation and regulation, such as the amygdala, hippocampus, and thalamus, might be affected by screen media use. For example, He et al. [18] found a negative correlation between social media use and the gray matter volume in the bilateral amygdala. In a study using longitudinal data, Takeuchi et al. [19] indicated that video game playing was associated with the delayed development of extensive brain regions, including the pallidum, putamen, hippocampus, caudate, and thalamus. More importantly, brain structural changes related to excessive screen media use (e.g., thinner hippocampus) were found to correlate with more severe externalizing problems [20,21]. Differential patterns of resting-state functional connectivity (RSFC) among these brain regions (e.g., prefrontal–limbic connectivity) also predicted future internalizing problems in adolescence [22,23]. As such, it may be beneficial to test whether the structural and functional alteration of subcortical brain regions were the potential mechanisms linking screen time and mental health from a holistic multi-modal perspective.

The present study aims to elucidate the longitudinal associations between screen media use, brain development, and mental health using a large-scale sample of early adolescents from the Adolescent Brain and Cognitive Development (ABCD) study [24]. Our first goal was to examine the distinct associations between various forms of screen media use (i.e., passive media, video games, social media, and total screen time) and mental health outcomes (e.g., internalizing and externalizing problems). Based on existing evidence, we hypothesized that time spent on different types of screen media would be associated with different mental health outcomes compared to total screen time. Our second goal was to test whether screen media use was associated with later mental health outcomes via structural and functional changes in subcortical brain regions (e.g., hippocampus, amygdala). We hypothesized that more screen media use would be associated with a volumetric change in subcortical brain regions and altered patterns of RSFC between cortical and subcortical brain regions, which, in turn, would be associated with severe mental health problems.

## 2. Materials and Methods

### 2.1. Participants

All data were drawn from the ABCD Study, which is an ongoing, 10-year longitudinal study that has recruited nearly 11,800 children (aged 9–10 years) at 21 sites throughout the United States, aiming to understand brain and behavior development through adolescence. Procedures were approved by individual sites’ institutional review boards, and all participants and legal guardians gave informed consent. Curated data from the baseline (T1) and year 2 of follow-up (T2) in the 4.0 release (https://abcdstudy.org/, accessed on 8 June 2022) were used in the present study because imaging data were collected every other year. Participants were excluded if (1) they had missing basic demographic data (i.e., sex, age, race/ethnicity, and family income), (2) they had missing screen time data, (3) they had missing magnetic resonance imaging (MRI) and resting-state functional magnetic resonance imaging (rsfMRI) data, and/or (4) their imaging data were recommended for exclusion by the ABCD analytic core [25]. The final sample consisted of 4619 participants (Male = 2396, see Table 1 for demographic information). The assessment methods were chosen based on several considerations, such as relevance to the study aims, feasibility and reliability, developmental appropriateness, psychometric properties, and compatibility with other large-scale studies.

### 2.2. Measures

Screen media use. Participants reported how many hours per weekday/weekend day they spend on six types of screen media, including television shows/movies, online videos, video games, texting, social media, and video chat. We further divided screen media use into three categories based on its interactivity and the time spent. Passive media use consists of watching television shows/movies and watching online videos. Social media use is a composite of texting, video chatting, and visiting social networking sites. Video game playing is considered the third type of screen media use.

MRI and rsfMRI Data. Imaging procedures of the ABCD study have been described in detail by Casey et al. [24]. Participants were scanned across 21 sites using the same protocols, and they completed four or five 5-minute resting-state scans with their eyes open to obtain at least 8 min of relatively low-motion data. Raw imaging data were preprocessed via the ABCD Data Analysis and Informatics Core using the standardized ABCD pipeline (for details and quality control procedures, see Hagler et al. [24]). We used measures of subcortical volume (*n* = 9, including the hippocampus, amygdala, caudate, putamen, pallidum, accumbens area, ventral diencephalon, thalamus proper, and brainstem; values for the right and left hemisphere were averaged) in our analyses. Using rsfMRI time courses, cortical network to subcortical connectivity was calculated based on the Gordon functional parcellation [26] for 12 predefined resting-state networks, including auditory network (AN), cingulo-opercular network (CON), cingulo-parietal network (CPN), default mode network (DMN), dorsal attention network (DAN), fronto-parietal network (FPN), retrosplenial temporal network (RTN), sensorimotor hand network (SHN), sensorimotor mouth network (SMN), salience network(SN), ventral attention network(VAN), and visual network (VN), leading to 96 variables. Therefore, our analyses included 104 imaging measures, all of which were drawn from Release 4.0 of the ABCD study.

Mental health outcomes. Raw scores from the Child Behavior Checklist [27] were used to measure syndromes of anxiety/depression, withdrawn behaviors, somatic complaints, thought problems, attention problems, rule-breaking behavior, aggressive behavior, social problems, internalizing problems, and externalizing problems. Symptoms were rated on a 3-point Likert-type scale of 0 (not true), 1 (somewhat or sometimes true), or 2 (very true or often true). The Child Behavior Checklist measures were completed by the parents/caregivers of the adolescents.

Covariates. Sex, age, race/ethnicity, and family income were included as covariates in the statistical analyses to adjust for potential confounding. Sex was a dichotomous variable (1 = male, 2 = female). Age was measured based on children’s age in years. The family income was converted to a categorical variable for different income brackets ranging from 1 (Less than USD 50,000) to 4 (USD 200,000 and greater).

### 2.3. Statistical Analyses

In the present study, we used linear mixed models (LMMs) in the R lme4 package [28] to test the association between screen media use and mental health outcomes, as well as brain measures. We covaried for sex, age, race/ethnicity, family income, and the baseline level of dependent variables in all analyses. We also covaried for mean framewise displacement during the resting-state scan (averaged across T1 and T2) in analyses predicting connectivity measures. To account for potential non-linear effects of motion, the quadratic effects of framewise displacement (FD^2^) were also incorporated into the analysis. The research sites were modeled as random effects to account for the nested structure of the data. Firstly, mental health measures in the second year were used as dependent variables (via a separate model) and three types of screen media use/total screen time at baseline as the independent variable. Secondly, subcortical volume or network to subcortical connectivity was used as a dependent variable. We controlled for multiple comparisons using the false discovery rate (*p*_FDR_ < .05). Standardized betas were also reported.

**Figure 1:**
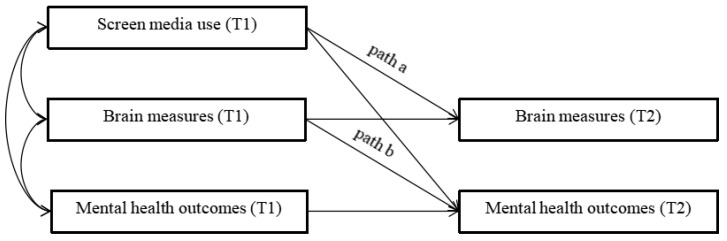
Conceptual half-longitudinal mediation model of the relationships between screen media use, brain measures, and mental health outcomes.

Exploratory analyses followed up any significant findings by examining whether screen media use was associated with later mental health outcomes via changes in subcortical structure/connectivity. The current study used a half-longitudinal mediation model [29] to examine the mediating role of brain measures between screen time and health measures (see the conceptual half-longitudinal mediation model in Figure 1). This model is based on general linear model assumptions, where both the path from exposure to mediator (path a) and the path from mediator to outcome (path b) were calculated using linear regression to estimate the indirect effect (path a × b) of exposure on the outcome through the mediator. Although mediation is typically tested with three-time points, Cole and Maxwell [29] demonstrated that the half-longitudinal mediation model can produce estimates similar to those obtained via a three-wave longitudinal mediation model. Bias-corrected bootstrap confidence interval levels were used to assess the significance of the indirect effect.

## 3. Results

### 3.1. Descriptive Statistics

Table 2 presents the descriptive statistics and correlation matrix of screen media use and mental health outcomes. Participants reported increased screen media use at T2 compared to T1 (*p*s < .001). Three different types of screen media use were moderately correlated with each other (*r*s < 0.5), which suggested that there is no perfect collinearity.

### 3.2. Associations between Screen Media Use and Mental Health Outcomes

As seen in Table 3, LMMs revealed that more total screen time was associated with increased rule-breaking behavior (*b* = .044, *SE* = .012, *p*_FDR_ = .003). For specific types of screen media, more passive media use was also associated with increased rule-breaking behavior (*b* = .038, *SE* = .012, *p*_FDR_ = .021), while more video game playing was associated with increased withdrawn/depressed symptoms (*b* = .041, *SE* = .013, *p*_FDR_ = .021). However, social media use was not associated with any mental health outcomes.

### 3.3. Associations between Screen Media Use and Brain Measures

**Figure 2:**
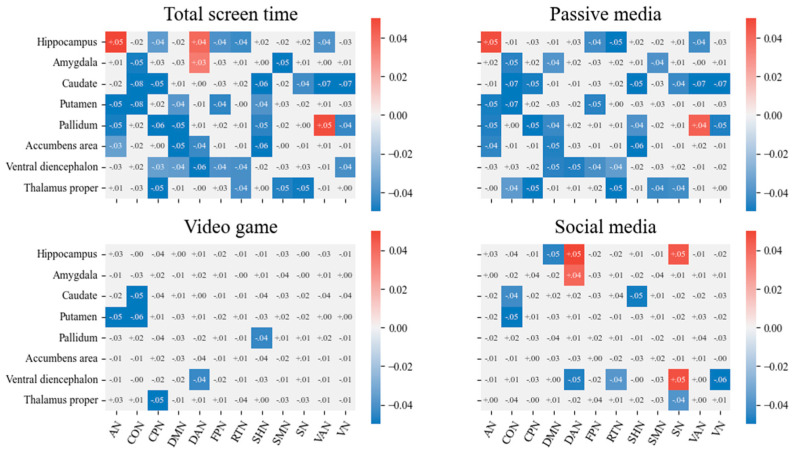
Associations between screen media use at T1 and cortical network to subcortical connectivity at T2. The heatmaps display the effects of specific types of screen media use on connectivity measures and the values are the standardized beta coefficients obtained from linear mixed models. Colored cells represent the model output reaching the significant level (*p*_FDR_ < .05, n comparisons = 96), and the *b*-value is displayed inside the cell. Sex, age, race/ethnicity, family income, mean framewise displacement (FD), quadratic effects of mean framewise displacement (FD^2^), and baseline level of dependent variables were included as covariates. AN, auditory network; CON, cingulo-opercular network; CPN, cingulo-parietal network; DMN, default mode network; DAN, dorsal attention network; FPN, fronto-parietal network; RTN, retrosplenial temporal network; SHN, sensorimotor hand network; SMN, sensorimotor mouth network; SN, salience network; VAN, ventral attention network; VN, visual network.

For subcortical volume, more social media use was associated with the reduced volume of the hippocampus (*b* = −.010, *SE* = .004, *p*_FDR_ = .039), caudate (*b* = −.007, *SE* = .003, *p*_FDR_ = .043), and thalamus proper (*b* = −.016, *SE* = .005, *p*_FDR_ = .022). However, other types of screen media use and total screen time were not associated with changes in the subcortical volume. For the cortical network to subcortical connectivity, higher screen media use was associated with reduced RSFC in a great number of subcortical areas (see Figure 2). For exploratory purposes, a comparative analysis was conducted on standardized beta coefficients for total screen time and three specific types of screen media use. The results showed significant differences between total screen time and video game playing (*t* = −3.493, *p*_Holm_ = .002, Cohen’s *d* = −.357), as well as social media use (*t* = −5.725, *p*_Holm_ < .001, Cohen’s *d* = −.584). However, no significant difference was observed between total screen time and passive media use (*t* = −.015, *p*_Holm_ = .988, Cohen’s *d* = −.001).

### 3.4. Mediation Analyses

To examine the mediating role of the cortical network to subcortical connectivity, mutually adjusted direct effects of screen media use and related brain measures on mental health outcomes were tested. However, the mediating effects of the cortical network on subcortical connectivity were not significant after controlling for multiple comparisons.

## 4. Discussion

The present study tested the longitudinal associations among screen media use, brain development, and mental health problems. The results showed that total screen time was correlated with rule-breaking behaviors. However, the associations with specific types of screen media use were different from total screen time. Specifically, increased withdrawn behaviors were associated with more video game playing but not total screen time. Social media use was not significantly associated with any mental health outcomes. In addition, the subcortical structure and RSFC matrix related to total screen time were significantly different from video games and social media. The findings suggested that the associations between screen media use, brain development, and mental health varied considerably depending on the type of activity. Therefore, future studies need to distinguish between different types of screen media, rather than simply combining all types of screen time.

Total screen time was not significantly associated with internalizing problems, which was inconsistent with previous studies [30]. One possible reason is that there may be some variables that could moderate the association between screen media use and mental health problems. For example, it has been found that girls, but not boys, who spend more time on screen media are more likely to have higher levels of internalizing problems [31]. A systematic review of moderating variables underlying the associations between screen media use and internalizing symptoms suggested that compared to other forms of screen media use, TV viewing was more weakly associated with internalizing symptoms [32]. The current findings can also be explained by protective factors associated with screen media use, such as resilience [33]. Another possible explanation is that the effects of screen media use on mental health problems might not be linear. Previous studies indicated that whereas both low and excessive screen media use were related to decreased psychological well-being, moderate use is related to increased psychological well-being [34]. Future studies need to examine the association between screen media use and mental health problems with even more granularity to test these hypotheses.

The present study showed that increased expression of withdrawn behaviors was associated with more video game playing but not with other types of screen media use. This is consistent with previous findings of an association between gaming addiction and withdrawn behaviors [35,36]. Adolescents who scored high on withdrawn behaviors are characterized by higher harm avoidance and lower reward dependence [37], which have been proven to be associated with the development of numerous problematic behaviors, including smartphone addiction [38] and problematic Internet use [39]. Moreover, Blasi et al. [40] indicated that game players may use video games as a coping strategy to escape from psychological difficulties, which could foster excessive engagement with video games and eventually lead to pathological gaming. Therefore, sophisticated modeling techniques should be used in future research to disentangle the possible bidirectional relationship between video game playing and withdrawn behaviors.

Consistent with our hypothesis, screen media use was associated with altered patterns of RSFC between the cortical network and subcortical brain regions. It is worth noting that compared to video games and social media, the RSFC pattern related to passive media use was not significantly different from total screen time. This finding suggested that combining all types of screen media use may obscure meaningful associations between specific screen media use and brain development. Moreover, there is neural evidence that compared to watching a pre-recorded gameplay video, the brain reward circuit was more sensitive to game-related successes when players were actively engaged in the gameplay. In contrast, the interaction between television viewing and social media was typically studied from a multitasking perspective. Pynta et al. [41] indicated that interacting on social media platforms (e.g., Twitter) while watching television can significantly enhance the brain activity measure of viewer engagement. Therefore, future studies should take the characteristics of different types of screen media into consideration to explore the associations between specific screen media use and developmental outcomes.

The results did not demonstrate that screen media use was associated with mental health problems via structural or functional changes in subcortical brain regions. However, neuroplasticity, i.e., the ability of the brain to reorganize its functional and structural connectivity according to intrinsic or extrinsic stimuli, has been demonstrated in many neuroimaging studies [42]. It is well acknowledged that neurons in the human cortex and subcortical areas are highly plastic, and different types of digital media use can influence various aspects of the human brain [43]. For example, touchscreen use can reshape the somatosensory cortex that processes tactile information from the fingertips [44]. Social media use can alter the brain anatomy and function of areas involved in emotional and social processing, especially in adolescents [45]. Various mental health problems, such as depression, schizophrenia, addiction, and PTSD, have been reported to be associated with abnormal changes in the brain [46]. The current study had limited data collection time points, which might have hidden significant findings. Conducting longitudinal studies with more brain-imaging scans at different developmental stages could help us to understand the impact of media use on brain development and its connection to mental health outcomes.

Although the longitudinal design and large sample could increase the generalizability of the results, several limitations should also be acknowledged. Firstly, only two time points during early adolescence were included in the current study. Thus, future studies need to examine how the associations between screen media use and developmental outcomes vary across ages. Secondly, the current study did not take into account the significant gender differences in screen media use, which have been reported in previous studies [47]. Future research is needed to investigate the impact of gender differences on the relationship between screen media use and developmental outcomes. Thirdly, the measures of screen media use focused solely on the amount, but not the content (e.g., violence) or other features. A previous study indicated that mental health issues, including depression, can be detected by analyzing social media content [48]. Therefore, future research is required to investigate whether the content of screen media could explain the associations between screen media use and mental health outcomes. Fourthly, our analyses of the structural and functional alteration focused on subcortical brain regions. The effects of screen media use on brain development are likely to be observed in other brain regions. Future work with more detailed assessments will contribute to a better understanding of such effects. Finally, this study may have a selective survival bias. Adolescents from low-income families were less likely to participate in follow-up data collection, which may weaken the effect of screen media use on developmental outcomes.

It is important to note that the observed effect sizes in this study were of modest magnitude, yet they possess the potential for substantive significance. The significance of these seemingly modest effect sizes is underscored by the recognition that their accumulation, when compounded over time and/or across diverse individuals, could yield consequential implications [49]. In light of the substantial size of the subject cohort examined in this investigation, even effect sizes characterized by extreme diminutiveness attain statistical significance. Consequently, further studies need to focus on the plausible amplification or attenuation of these effects during the developmental trajectory of an adolescent. By understanding the unique characteristics and impacts associated with specific types of screen media, policymakers can develop targeted strategies that promote healthy screen habits and safeguard the well-being of the younger generation.

## 5. Conclusions

Using longitudinal data from the ABCD Study, the present study distinguished between different types of screen media use, with each shown to have different associations with brain development measures and mental health problems. These findings suggested that not all screen time is created equal, and more research is needed to examine the effects of screen media use on neurodevelopmental processes and mental health problems during adolescence.

## Figures and Tables

**Table 1:** Demographic information.

Characteristics	Original Sample	Final Sample
	*N* or Mean ± SD	
*N* Total (*n* Male)	11876 (6192)	4619 (2396)
Age, Years	9.48 ± 0.51	9.50 ± 0.51
Race/ethnicity		
White	6208 (52.3%)	2773 (60.0%)
Black	1796 (15.1%)	464 (10.0%)
Hispanic	2411 (20.3%)	857 (18.6%)
Asian	228 (1.9%)	65 (1.4%)
Other	1233 (10.4%)	460 (10.0%)
Family income (past 12 months)		
Less than $50,000	3223 (27.1%)	1198 (25.9%)
USD 50,000 through USD 99,999	3071 (25.8%)	1415 (30.6%)
USD 100,000 through USD 199,999	3314 (27.9%)	1490 (32.3%)
USD 200,000 and greater	1250 (10.5%)	516 (11.2%)
Screen media use (hours)		
Passive media	2.41 ± 1.8	2.26 ± 1.7
Video game	1.08 ± 1.1	1.00 ± 1.1
Social media	0.56 ± 1.2	0.47 ± 1.0
Total screen time	4.03 ± 3.2	3.72 ± 2.9

Note. Demographic information was measured at T1.

**Table 2:** The descriptive statistics and correlation matrix of screen media use and mental health outcomes.

Variable	1	2	3	4	5	6	7	8	Mean	SD
Screen media use										
1. Passive media, T1	—								2.26	1.71
2. Passive media, T2	.38 *	—							3.39	3.26
3. Video game, T1	.46 *	.26 *	—						1.00	1.06
4. Video game, T2	.28 *	.54 *	.39 *	—					2.05	2.91
5. Social media, T1	.33 *	.20 *	.24 *	.13 *	—				0.47	0.99
6. Social media, T2	.20 *	.48 *	.09 *	.33 *	.32 *	—			1.74	3.72
7. Total screen time, T1	.88 *	.39 *	.72 *	.35 *	.63 *	.26 *	—		3.72	2.88
8. Total screen time, T2	.36 *	.84 *	.29 *	.75 *	.28 *	.79 *	.42 *	—	7.17	7.86
Mental health outcomes										
9. Anxiety/depression, T1	.01	.01	.02	.00	.00	−.01	.01	.00	2.46	2.96
10. Anxiety/depression, T2	−.02	.02	−.02	−.03	.00	.00	−.02	.00	2.31	2.93
11. Withdrawn, T1	.04	.06 *	.05	.07 *	−.01	.01	.04	.05 *	0.94	1.56
12. Withdrawn, T2	.04	.08 *	.06 *	.04	.01	.03	.05	.06 *	1.17	1.87
13. Somatic, T1	.07 *	.08 *	.01	.01	.02	.05	.05 *	.06 *	1.47	1.88
14. Somatic, T2	.06 *	.08 *	.02	.02	.03	.04	.05 *	.06 *	1.38	1.87
15. Social, T1	.11 *	.12 *	.06 *	.09 *	.05 *	.05 *	.10 *	.11 *	1.43	2.07
16. Social, T2	.08 *	.11 *	.06 *	.07 *	.05 *	.04	.08 *	.09 *	1.21	1.96
17. Thought, T1	.06 *	.07 *	.08 *	.06 *	.02	.01	.07 *	.05 *	1.53	2.02
18. Thought, T2	.06 *	.08 *	.07 *	.05	.02	.02	.07 *	.06 *	1.38	1.99
19. Attention, T1	.12 *	.11 *	.13 *	.14 *	.05	.01	.13 *	.10 *	2.74	3.28
20. Attention, T2	.11 *	.12 *	.13 *	.15 *	.04	.04	.13 *	.12 *	2.55	3.19
21. Rule breaking, T1	.14 *	.14 *	.11 *	.15 *	.12 *	.09 *	.17 *	.16 *	1.07	1.72
22. Rule breaking, T2	.13 *	.15 *	.11 *	.14 *	.10 *	.11 *	.16 *	.16 *	0.99	1.76
23. Aggressive, T1	.10 *	.10 *	.08 *	.10 *	.06 *	.05	.11 *	.10 *	3.01	4.10
24. Aggressive, T2	.09 *	.11 *	.07 *	.10 *	.06 *	.08 *	.10 *	.12 *	2.73	3.82
25. Internalizing, T1	.05	.05 *	.03	.03	.00	.02	.04	.04	4.87	5.23
26. Internalizing, T2	.02	.06 *	.02	.01	.01	.02	.03	.04	4.86	5.50
27. Externalizing, T1	.12 *	.12 *	.10 *	.12 *	.08 *	.06 *	.13 *	.12 *	4.08	5.49
28. Externalizing, T2	.11 *	.13 *	.09 *	.12 *	.08 *	.09 *	.12 *	.14 *	3.72	5.25

Note. *N* = 4619, * *p* < .001. Conditioned on variables: sex, age, race/ethnicity, and family income. T1 = baseline, T2 = the year 2 follow-up.

**Table 3:** Associations between screen media use at T1 and mental health outcomes/subcortical volume at T2.

	Passive Media			Video Game			Social Media			Total Screen Time		
	*b*	*p*	*p*_FDR_	*b*	*p*	*p*_FDR_	*b*	*p*	*p*_FDR_	*b*	*p*	*p*_FDR_
Mental health outcomes												
Anxiety/depression	−.021	.080	.226	−.013	.286	.477	−.001	.951	.951	−.018	.143	.239
Withdrawn/depression	.009	.477	.530	.041	.002	.021 *	.002	.874	.951	.021	.111	.223
Somatic	.011	.374	.530	.012	.379	.486	.010	.410	.683	.015	.253	.316
Social	.009	.440	.530	.015	.226	.452	.016	.187	.622	.017	.172	.245
Thought	.019	.113	.226	.028	.026	.086	.011	.335	.671	.026	.035	.118
Attention	.023	.031	.154	.020	.069	.173	.007	.486	.695	.024	.028	.118
Rule breaking	.038	.002	.021 *	.030	.018	.086	.029	.015	.146	.044	.000	.003 *
Aggressive	.011	.327	.530	.002	.875	.875	.011	.299	.671	.011	.311	.345
Internalizing	−.007	.572	.572	.009	.441	.490	.004	.713	.892	.001	.946	.946
Externalizing	.018	.108	.226	.010	.389	.486	.015	.157	.622	.020	.075	.187
Subcortical volume												
Hippocampus	−.008	.035	.140	.002	.692	.923	−.010	.010	.039 *	−.008	.041	.109
Amygdala	.000	.951	.951	−.004	.498	.797	−.004	.461	.527	−.003	.587	.587
Caudate	.000	.885	.951	−.002	.423	.797	−.007	.016	.043 *	−.004	.219	.438
Putamen	−.002	.591	.945	.000	.925	.925	−.003	.455	.527	−.003	.533	.587
Pallidum	−.021	.011	.090	−.017	.056	.226	−.010	.242	.388	−.023	.008	.068
Accumbens area	−.008	.282	.564	.001	.873	.925	−.009	.233	.388	−.008	.310	.497
Ventral diencephalon	.002	.748	.951	.012	.034	.226	−.002	.681	.681	.004	.416	.555
Thalamus proper	−.008	.130	.346	−.005	.420	.797	−.016	.003	.022 *	−.013	.024	.094

Note. The table reports standardized beta coefficients and uncorrected and corrected *p* values. * represents the model output reaching the significant level (*p*_FDR_ < .05). Sex, age, race/ethnicity, family income, and baseline level of dependent variables were included as covariates.

## Data Availability

Data used in the preparation of this article were obtained from the Adolescent Brain Cognitive Development (ABCD) Study (https://abcdstudy.org, accessed on 8 June 2022), held in the NIMH Data Archive (NDA). This is a multisite, longitudinal study designed to recruit more than 10,000 children aged 9–10 and follow them over 10 years into early adulthood. The ABCD Study^®^ is supported by the National Institutes of Health and additional federal partners under award numbers U01DA041048, U01DA050989, U01DA051016, U01DA041022, U01DA051018, U01DA051037, U01DA050987, U01DA041174, U01DA041106, U01DA041117, U01DA041028, U01DA041134, U01DA050988, U01DA051039, U01DA041156, U01DA041025, U01DA041120, U01DA051038, U01DA041148, U01DA041093, U01DA041089, U24DA041123, and U24DA041147. A full list of supporters is available at https://abcdstudy.org/federal-partners.html, accessed on 8 June 2022. A listing of participating sites and a complete listing of the study investigators can be found at https://abcdstudy.org/consortium_members/, accessed on 8 June 2022. ABCD consortium investigators designed and implemented the study and/or provided data but did not necessarily participate in the analysis or writing of this report. This manuscript reflects the views of the authors and may not reflect the opinions or views of the NIH or ABCD consortium investigators. The ABCD data repository grows and changes over time. The ABCD data used in this report came from https://doi.org/10.15154/1523041, accessed on 8 June 2022.
